# Subclinical Atrial Fibrillation Prediction in Patients with CIED by a Novel Deep Learning Framework

**DOI:** 10.3390/jcdd13010018

**Published:** 2025-12-30

**Authors:** Yongying Lan, Chengze Lin, Ning Zhang, Qing Cao, Qi Jin, Qingzhi Luo, Yue Wei, Yangyang Bao, Changjian Lin, Wenqi Pan, Kang Chen, Liqun Wu, Yun Xie

**Affiliations:** 1 Department of Cardiovascular Diseases, Ruijin Hospital, Shanghai Jiao Tong University School of Medicine, Shanghai 200025, China; lanyongying@sjtu.edu.cn (Y.L.); lcz0824@sjtu.edu.cn (C.L.); zn11476@rjh.com.cn (N.Z.); cq30553@rjh.com.cn (Q.C.); jinqi127@163.com (Q.J.); 18817821284@163.com (Q.L.); weiyue0720@gmail.com (Y.W.); oliverbao@icloud.com (Y.B.); linchangjian222@126.com (C.L.); drpanwenqi@163.com (W.P.); chenkang1978@gmail.com (K.C.); 2 Shanghai Artificial Intelligence Laboratory, Shanghai 200233, China

**Keywords:** subclinical atrial fibrillation, deep learning, Kolmogorov–Arnold network, explainable AI, clinical decision support tool

## Abstract

Background: Subclinical atrial fibrillation (SCAF), a key risk factor for cryptogenic stroke, is difficult to predict with current tools. This study aimed to develop a novel deep learning framework, ResKAN-Attention, using only routine clinical data to predict SCAF in patients with cardiac implantable electronic device (CIED). Methods: In this retrospective study, the ResKAN-Attention model was developed using 27 routine parameters from 124 CIED patients without prior AF. This framework features a dual-path architecture combining a Kolmogorov–Arnold Network (KAN) with a traditional multilayer perceptron, fused via a cross-attention mechanism. The model’s performance was evaluated against common baselines using five-fold cross-validation, while its decision-making process was assessed through interpretability analysis. A clinically applicable risk scoring system was subsequently derived via knowledge distillation. Results: Over a 12-month follow-up period, SCAF occurred in 31.5% of patients (39/124). The ResKAN-Attention model significantly outperformed all baseline models, achieving a mean AUC of 0.837 in cross-validation and 0.788 in external validation. Interpretability analysis identified left atrial diameter (LAD), gender, lactate dehydrogenase, BMI, and hypertension as top predictors. The simplified risk score exhibited excellent predictive power (AUC 0.882), retaining 99.1% of the complex model’s performance on the fifth fold validation set. Conclusions: The ResKAN-Attention model demonstrated promising preliminary results for SCAF prediction with enhanced interpretability. The distilled risk score provided a potential method for early risk stratification in clinical settings, demonstrating that advanced artificial intelligence (AI) can effectively predict complex cardiovascular events using readily available data.

## 1. Introduction

SCAF, characterized by asymptomatic rapid atrial arrhythmias, is increasingly recognized as a significant cause of cryptogenic stroke [1,2]. Previous studies have revealed a high prevalence of atrial high-rate episodes (AHREs) in elderly patients recorded by CIED [3]. Most AHREs are caused by atrial fibrillation, while device-detected SCAF has been proven to be a predictor of future clinical AF [4]. However, the lack of reliable clinical tools to predict SCAF occurrence, together with the impracticality of long-term wearable monitoring in asymptomatic populations, hinders timely intervention and outcome improvement. Consequently, developing a simple and effective clinical prediction model for early identification of SCAF is critical.

The emergence of AI technologies offers new possibilities for significantly expanding the diagnostic and prognostic potential of clinical tools [5]. In recent years, the application of deep learning in medicine has advanced significantly, evolving from multilayer perceptron (MLP) to residual networks (ResNet), Transformers, and the recently introduced KAN. As a novel architecture, the KAN places learnable activation functions on network edges, replacing fixed functions, and abandoning linear weight matrices in favor of parameterized univariate spline functions. This enhances nonlinear modeling capabilities, enabling the precise capture of complex data patterns [6].

In this study, we developed a new deep learning framework, termed ResKAN-Attention, which integrates demographic, echocardiographic, and biochemical parameters to predict the risk of SCAF. We further distilled the model into a simplified score via knowledge distillation and externally validated it in an independent cohort, demonstrating its strong potential for practical clinical application.

## 2. Methods

### 2.1. Patient Enrollment and Clinical Data Collection

**Figure 1:**
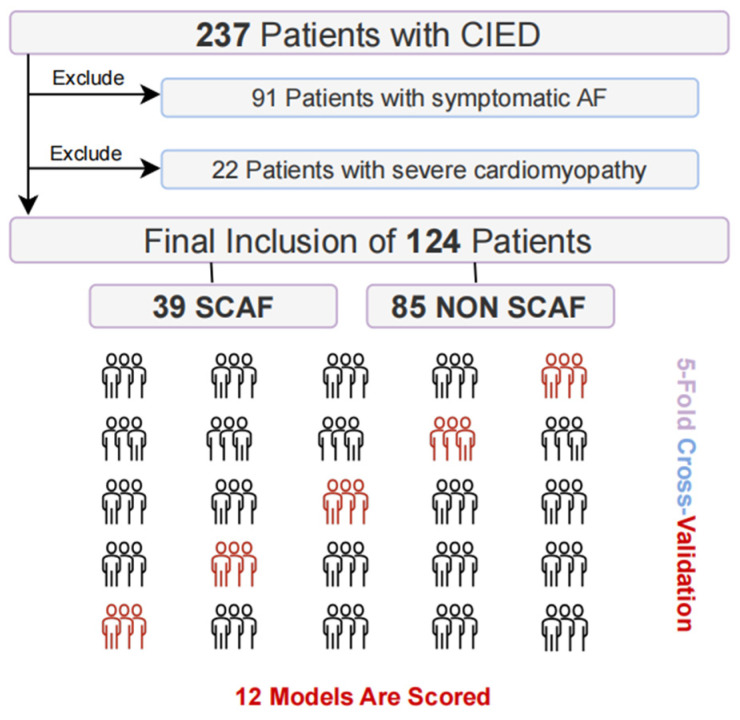
Flowchart illustrating the patient selection process for this study.

This study retrospectively enrolled consecutive patients who underwent permanent dual-chamber pacemaker implantation at our institution from August 2023 to February 2024 and completed a routine 12-month follow-up in the CIED clinic. A total of 237 patients were initially enrolled in this study. Follow-up CIED clinic visits were scheduled at 1, 3, 6, and 12 months post-implantation. All arrhythmia data recorded by CIED were collected during visits. Any patient with a CIED-detected AHRE with irregular A-A intervals lasting >30 s was considered to have SCAF. AF-related symptoms were also collected. Ninety-one patients who were previously diagnosed with paroxysmal AF or developed symptoms related to AHRE after CIED implantation were excluded from the study. Twenty-two patients with significant cardiomyopathy who received cardiac resynchronization therapy (CRT) or implantable cardioverter-defibrillator (ICD) implantation were also excluded from the final analysis (Figure 1). Clinical features before CIED implantation of the remaining 124 patients were then investigated. A total of 27 parameters were collected, including both demographic and clinical characteristics. Demographic characteristics included gender, age, and body mass index (BMI). Clinical characteristics encompassed medical history (hypertension, diabetes, coronary heart disease, valvular heart disease), biochemical markers (NT-proBNP, hsTnI, CRP, Myoglobin, CK-MB, lactate dehydrogenase (LDH), Lp(a), LDL-C, serum creatinine, eGFR calculated by simplified MDRD formula), standard transthoracic echocardiographic parameters (left atrial diameter [LAD], left ventricular ejection fraction [LVEF], left ventricular end-diastolic diameter [LVEDD], left ventricular end-diastolic volume [LVEDV], interventricular septal [IVS] thickness, left ventricular posterior wall [LVPW] thickness), as well as tissue Doppler imaging parameters (septal E/e′, lateral E/e′).

### 2.2. Data Preprocessing

To address missing data and class imbalance, we employed IterativeImputer for missing value imputation [7], followed by log1p transformation for skewed features (NT-proBNP, CRP) and StandardScaler for normalization [8]. To prevent data leakage, all preprocessing steps were fitted exclusively on training data within each cross-validation fold. Synthetic minority over-sampling technique (SMOTE) was applied only to training sets to balance the SCAF-positive and SCAF-negative classes [9]. Detailed preprocessing parameters are listed in Table S1.

### 2.3. Model Development

**Figure 2:**
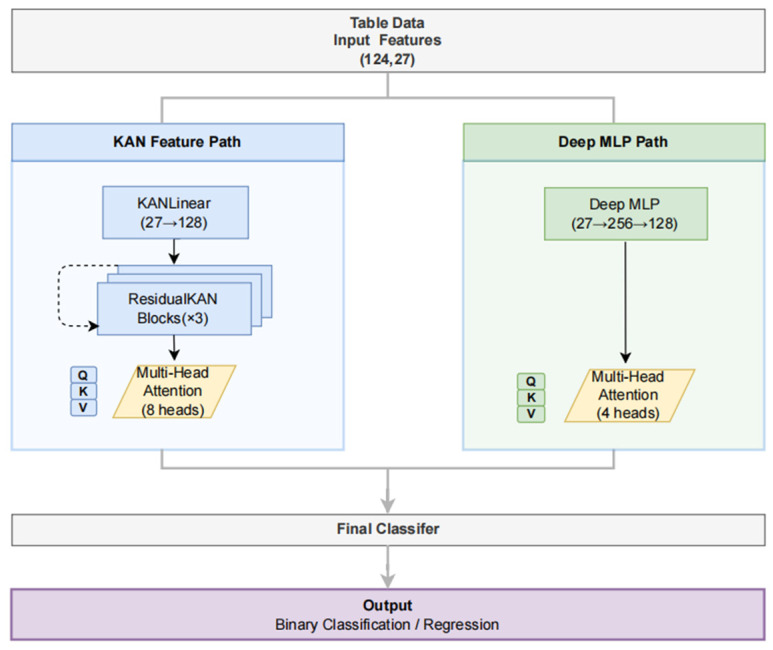
Schematic diagram of the ResKAN-Attention model architecture.

The ResKAN-Attention model employs a dual-pathway architecture comprising a ResKAN pathway utilizing Kolmogorov–Arnold Network layers for flexible nonlinear modeling and an MLP pathway for conventional feature extraction. Outputs from both pathways are integrated through a cross-attention fusion module to generate the final SCAF risk prediction [10]. The complete architecture is illustrated in Figure 2.

### 2.4. Baseline Models

To comprehensively evaluate the performance of the proposed ResKAN-Attention model, we constructed a series of classic models as baselines. These include traditional machine learning models: Logistic Regression, XGBoost, Random Forest, CatBoost, LightGBM, K-Nearest Neighbors (KNN), Gaussian NB and Support Vector Machine (SVM). Standard deep learning models: MLP, ResNet, Transformer-based FT-Transformer model [11], and KAN. The hyperparameter search spaces for all baseline models are detailed in Table S2.

### 2.5. Model Training and Evaluation

All deep learning models were implemented in PyTorch and optimized using AdamW [12]. Early stopping was applied to prevent overfitting. Five-fold cross-validation was performed, with AUC as the primary metric and accuracy, precision, recall, and F1 score as secondary metrics. Hyperparameters were optimized via grid search. Statistical significance between models was assessed using paired-samples t-tests and Cohen’s d effect sizes [13]. All experiments were conducted in Python 3.9; the ResKAN-Attention model hyperparameters are presented in Table S3.

### 2.6. Model Interpretability Analysis

SHAP (SHapley Additive exPlanations) was applied to identify global feature importance and LIME (Local Interpretable Model-agnostic Explanations) for patient-specific interpretations [14,15]. Additionally, activation patterns in the KAN layers were visualized to reveal the nonlinear relationships learned by the model, providing further insight into key clinical predictors of SCAF.

### 2.7. Knowledge Distillation for Clinical Risk Scoring

To enhance clinical applicability, knowledge distillation was employed to transfer knowledge from the ResKAN-Attention teacher model to a logistic regression student model [16]. The distillation process focused on the top 10 features identified by SHAP analysis. Specific distillation parameters and loss functions are provided in Table S4. The resulting student model converts the linear risk score into a SCAF probability, with a threshold of 0.5 for high-risk classification.

### 2.8. External Validation

We prospectively enrolled 35 patients who underwent permanent dual-chamber pacemaker implantation at our institution between December 2024 and March 2025, with a SCAF prevalence of 28.6% (10/35 patients). All patients routinely completed a 6-month follow-up visit in the CIED clinic, ensuring comprehensive post-implantation monitoring. Clinical data were collected in alignment with the primary cohort, encompassing the same 27 parameters, and preprocessed consistently with the training dataset to maintain comparability.

## 3. Results

### 3.1. Patient Characteristics

According to the pacemaker recordings, SCAF was detected in 39 out of 124 patients (31.5%), while the remaining 85 patients (68.5%) comprised the non-SCAF group. The mean age was 72.9 ± 7.8 years in the SCAF group and 71.5 ± 12.0 years in the non-SCAF group, with male proportions of 35.9% and 63.5%, respectively. The proportion of patients with a history of severe mitral valve disease was significantly higher in the SCAF group than in the non-SCAF group (12.8% vs. 1.2%), while no significant difference was found in all the other comorbidities between the two groups. For biochemical markers, NT-proBNP level was significantly higher in the SCAF group (926.2 ± 1271.3 pg/mL) compared to the non-SCAF group (393.8 ± 620.0 pg/mL). Left atrial diameter (LAD) measured through transthoracic echocardiography was 43.9 ± 5.0 mm in the SCAF group and 40.3 ± 4.1 mm in the non-SCAF group, which was statistically significant. The detailed data and comparison of clinical characteristics between the two groups are shown in Table 1. During the 12-month follow-up, no stroke or systemic embolic events were documented in either group.

### 3.2. Model Performance

To explore the best model for the prediction of SCAF, both common machine learning models and deep learning models were attempted. Among traditional machine learning models, the SVM model exhibited the best overall performance, with an AUC of 0.753 ± 0.134, accuracy (ACC) of 0.742 ± 0.071, and F1 score of 0.624 ± 0.093. In the deep learning baseline models, KAN model performed best, achieving an AUC of 0.825 ± 0.037, ACC of 0.735 ± 0.058, and F1 score of 0.589 ± 0.096 (Table 2).

**Figure 3:**
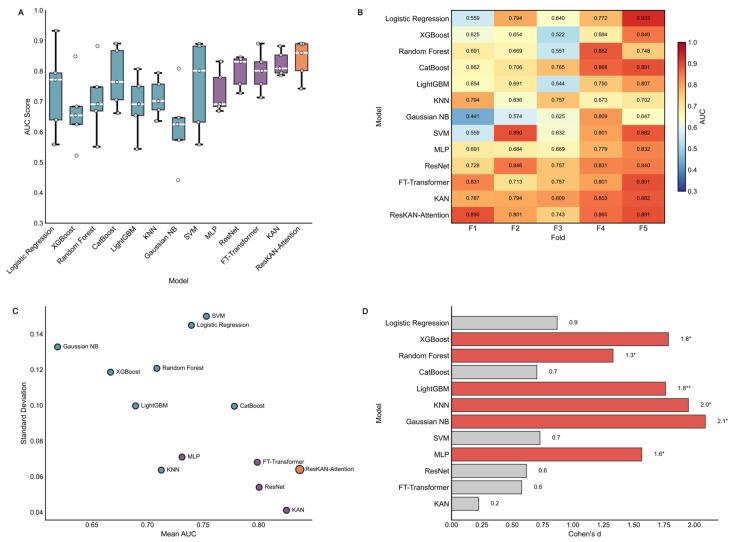
Performance comparison of the ResKAN-Attention model with common models. (**A**) Boxplot comparing the distribution of Area Under the Curve (AUC) scores. The central line, box, and whiskers represent the median, interquartile range, and minimum/maximum values, respectively. (**B**) Heatmap of the fold-specific AUC score for each model. (**C**) Scatterplot of mean AUC versus standard deviation. (**D**) Bar chart of Cohen’s d. Asterisks denote statistical significance.

The performance of the ResKAN-Attention model was then analyzed. In the five-fold cross-validation, the model achieved the highest average AUC of 0.837 ± 0.057 (Table 2, Figure 3A,B) with low standard deviation, suggesting its accuracy and stability (Figure 3C). We then analyzed the effect size of the performance difference using Cohen’s d. A large effect size was revealed compared to most baseline models, further indicating the superiority of the model (Figure 3D).

**Figure 4:**
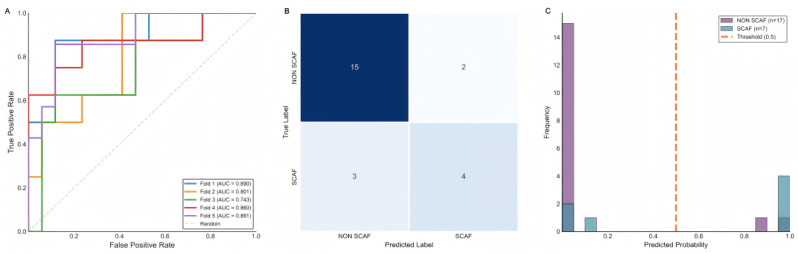
Performance of the ResKAN-Attention model in the fifth cross-validation fold. (**A**) ROC curves from five-fold cross-validation of the ResKAN-Attention model. (**B**) Confusion matrix for the fifth cross-validation fold, detailing true and false classifications. (**C**) Histogram of predicted probabilities.

The peak performance of the ResKAN-Attention model was observed in the fifth cross-validation fold. In this specific fold, it achieved a validation set AUC of 0.891, demonstrating excellent discriminative ability (Figure 4A). The corresponding confusion matrix detailed the classification results, correctly identifying 4 true positives and 15 true negatives (Figure 4B). The histogram of predicted probabilities for this fold further illustrates the model’s capacity to effectively separate SCAF-positive from SCAF-negative cases (Figure 4C).

In the external validation cohort, the ResKAN-Attention model demonstrated robust generalization, achieving an AUC of 0.788 (Supplementary Figure S3). Additional performance metrics included accuracy of 0.800, precision of 0.667, recall of 0.600, and F1-score of 0.632.

### 3.3. Model Interpretability Analysis

To elucidate the decision-making process and further explore the potential risk factors of SCAF, interpretability analysis was conducted at three distinct levels.

**Figure 5:**
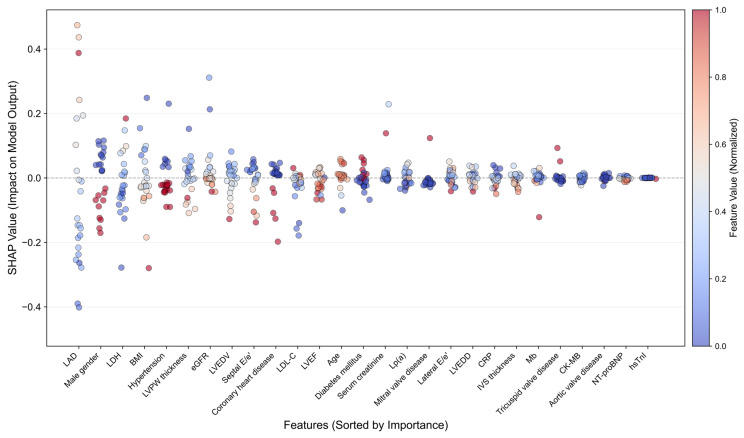
Global feature importance derived from SHapley Additive exPlanations analysis of the ResKAN-Attention model.

Global feature importance analysis using SHAP was initially applied to identify the key predictors of SCAF (Figure 5). Among the 27 features, LAD exhibited the strongest predictive contribution, with its SHAP value significantly surpassing that of other features. Following LAD, the most influential features were gender, LDH, BMI, and hypertension.

We applied the LIME method to generate local attribution explanations in several representative patients, elucidating the model’s case-specific prediction logic (Supplementary Figure S1). Elevated LAD was shown to be the dominant factor in most predictions, while the contribution of other parameters seemed to vary in different cases. Active neurons were also extracted from the KAN input layer to visualize their responses in comparison with the top eight most important features identified by SHAP analysis (Supplementary Figure S2). Each single input parameter elicited diverse responses in different neurons, as no specific tendency suitable for all the clinical features could be identified. All these analyses suggested that SCAF prediction was complex and multifactorial, demonstrating the necessity of applying AI-assisted models.

### 3.4. Development and Validation of Clinical SCAF Risk Scoring

In an attempt to achieve a simplified, clinically applicable predictive tool for SCAF, we transferred the predictive capability of the complex ResKAN-Attention model to a simplified logistic regression model based on the top 10 key clinical features selected according to their SHAP contribution scores, with the assistance of knowledge distillation. The linear SCAF Risk Score is calculated as follows:

$$\begin{array}{ll} SCAF\;Risk\;Score & =0.407314\times LAD(mm)-1.810896\times Male\;gender\\ & +\;2.026237\times ln(“LDH(IU/L)”+1)-0.128719\times “BMI(kg/m^{2})”\\ & -\;0.571839\times “Hypertension”-0.439865\times “LVPW\;thickness(mm)”\\ & -\;0.000892\times “eGFR(mL/min/1.73\;m^{2})”+0.005358\times “SeptalE/e^{\prime }”\\ & -\;0.023019\times “LVEDV(mL)”-1.335999\times “Coronary\;heart\;disease”\\ & -\;17.815156 \end{array}$$

The probability of SCAF was subsequently calculated. On the fifth-fold validation set, this formula achieved an AUC of 0.882, closely aligning with the teacher model’s AUC of 0.891, with an AUC retention rate of 99.1%. The correlation coefficient between the predicted probabilities of the two models was 0.9091, with a mean absolute error of only 0.1164, demonstrating the effectiveness of the distillation process. The formula achieved an AUC of 0.788 on the external validation set (Supplementary Figure S3).

These results indicate that the simplified clinical risk score maintains high predictive performance while significantly enhancing its applicability in clinical settings.

## 4. Discussion

In this study, we introduced the ResKAN-Attention model, a novel deep learning framework designed to predict the occurrence of SCAF within one year using routinely collected clinical parameters. By leveraging a dual-path architecture that integrates the KAN with MLP and a cross-attention fusion mechanism, our model demonstrated promising predictive performance compared to existing models in this pilot cohort. The incorporation of SHAP and LIME techniques enhances interpretability, providing both global and individual-level explanations of predictions. Furthermore, through knowledge distillation, we derived a simplified, clinically applicable formula that maintains predictive accuracy while being practical for resource-constrained settings. These findings suggest that this approach may help address gaps in SCAF prediction.

Electrocardiogram (ECG) data have been widely applied for the prediction of AF. Although typical ECG-based models utilizing standard 12-lead ECG or Holter monitoring as data input have shown high accuracy [5,17,18], they face limitations in monitoring continuity and are especially impractical for the detection of SCAF. Wearable devices employing photoplethysmography (PPG) have emerged as an alternative, yet their signal instability and impracticality for population-wide monitoring pose significant challenges [19,20]. In contrast, the ResKAN-Attention model utilizes widely available clinical parameters, such as echocardiographic measurements and basic biochemical markers. As all of the parameters could be routinely collected in elderly patients, our approach attempted to offer broader clinical applicability and reduce dependence on specialized monitoring devices. However, risk stratification may vary over time, which requires frequent adjustment to ensure the credibility of the model [21]. Whether our model could maintain its prediction capability after 12 months and a better time interval for the reassessment of all the parameters remains to be clarified in future studies.

The interpretability analysis not only elucidated the feature importance underlying AI algorithm predictions but also provided clinically relevant insights into SCAF. Firstly, several key findings were consistent with established clinical knowledge, which reinforced the biological plausibility of our model. LAD emerged as the most critical predictor of SCAF across all analyses, aligning with the clinical understanding of atrial pressure and remodeling as key drivers of AF pathogenesis [22]. LDH, another important predictor in our model, has also been reported to be positively related to AF occurrence in several studies [23]. Conversely, some results challenge conventional clinical assumptions. For instance, while epidemiological data suggest a higher prevalence of symptomatic AF in males [24], our model identified higher SCAF risk in females. This discrepancy may reflect differences in symptom tolerance, as females potentially under-report AF-related symptoms, leading to a higher proportion of undetected SCAF. Similar unexpected findings were observed for BMI and hypertension [25], suggesting that single-factor predictions may be insufficient for SCAF. These observations underscore the necessity of multifactorial, data-driven models like ResKAN-Attention to capture complex interactions among clinical variables. NT-proBNP, despite being significantly elevated in the SCAF group, was not among the final top 10 predictors. This suggests that NT-proBNP may primarily reflect the atrial pressure state at the time of measurement, with limited predictive efficacy for long-term arrhythmic events. All these findings emphasize the value of comprehensive feature integration in this model. Further studies are needed to validate these hypotheses and elucidate their underlying mechanisms.

The ResKAN-Attention model was built on recent advancements in AI, particularly the KAN architecture, which offers distinct advantages over traditional neural networks. By placing learnable activation functions on network edges and replacing linear weight matrices with parameterized univariate spline functions, KAN enhances nonlinear modeling capabilities [6]. This enables the model to capture intricate patterns in complex, feature-diverse datasets, such as those encountered in cardiology. The dual-path architecture, combining KAN with MLP and a cross-attention mechanism, effectively integrates linear and nonlinear data relationships, resulting in a more robust and accurate predictive framework compared to single-model architectures like ResNet or Transformer models.

Despite these promising results, our study has several limitations. As a single-center study, the model was developed and validated using data from one institution, which may limit its generalizability. The study population was restricted to patients with CIED, who were at higher risk of AHRE. The model’s performance in other patient groups remains to be validated. Future multicenter studies in diverse populations and with prospective validation are essential to confirm the model’s broader applicability and robustness.

## 5. Conclusions

In conclusion, the ResKAN-Attention model exhibited promising preliminary results for SCAF prediction with enhanced interpretability and potential clinical applicability. By utilizing routine clinical parameters and a novel AI architecture, our model provided a framework for SCAF risk stratification. This framework represents a potential tool that warrants further investigation for improving the early detection and management of SCAF in clinical practice.

## Figures and Tables

**Table 1:** Baseline characteristics of study population.

Characteristics	Total	SCAF Group	Non-SCAF Group	*p*-Value
	(N = 124)	(N = 39, 31.5%)	(N = 85, 68.5%)	
Demographics				
Age (years)	72.0 ± 10.8	72.9 ± 7.8	71.5 ± 12.0	0.43
Male gender	68 (54.8)	14 (35.9)	54 (63.5)	0.006
BMI (kg/m^2^)	24.0 ± 3.7	23.6 ± 4.2	24.1 ± 3.4	0.47
Comorbidities				
Hypertension	84 (67.7)	24 (61.5)	60 (70.6)	0.41
Diabetes mellitus	38 (30.6)	10 (25.6)	28 (32.9)	0.53
Coronary heart disease	33 (26.6)	6 (15.4)	27 (31.8)	0.08
Aortic valve disease	6 (4.8)	2 (5.1)	4 (4.7)	1
Mitral valve disease	6 (4.8)	5 (12.8)	1 (1.2)	0.01
Tricuspid valve disease	9 (7.3)	5 (12.8)	4 (4.7)	0.14
Biochemical Parameters				
NT-proBNP (pg/mL)	564.0 ± 911.1	926.2 ± 1271.3	393.8 ± 620.0	0.02
hsTnI (pg/mL)	135.7 ± 1224.1	25.9 ± 56.3	183.3 ± 1465.3	0.33
CRP (mg/L)	10.2 ± 23.2	4.0 ± 6.2	13.1 ± 27.3	0.03
Myoglobin (ng/mL)	31.8 ± 21.1	29.3 ± 14.6	32.9 ± 23.3	0.3
CK-MB (ng/mL)	1.8 ± 1.3	1.8 ± 1.5	1.8 ± 1.3	0.98
LDH (IU/L)	218.3 ± 73.0	224.1 ± 50.5	215.6 ± 81.3	0.5
Lp(a) (g/L)	0.2 ± 0.2	0.3 ± 0.2	0.2 ± 0.2	0.33
LDL-C (mmol/L)	2.3 ± 0.9	2.4 ± 0.9	2.2 ± 0.9	0.29
Serum creatinine (µmol/L)	83.1 ± 25.6	83.5 ± 29.8	82.9 ± 23.6	0.92
eGFR (mL/min/1.73 m^2^)	74.9 ± 17.8	71.7 ± 19.1	76.4 ± 17.1	0.2
Echocardiographic Parameters				
Left atrial diameter (mm)	41.5 ± 4.7	43.9 ± 5.0	40.3 ± 4.1	<0.001
LVEF (%)	65.2 ± 7.0	65.2 ± 5.0	65.3 ± 7.8	0.99
LVEDD (mm)	49.5 ± 6.0	48.2 ± 5.2	50.1 ± 6.2	0.08
LVEDV (mL)	118.2 ± 32.1	112.4 ± 28.6	120.7 ± 33.4	0.18
IVS thickness (mm)	10.1 ± 1.7	10.0 ± 1.9	10.1 ± 1.7	0.89
LVPW thickness (mm)	9.1 ± 1.1	8.9 ± 1.3	9.2 ± 1.1	0.34
Septal E/e′	12.4 ± 6.5	13.8 ± 6.5	11.8 ± 6.5	0.14
Lateral E/e′	9.0 ± 3.6	9.9 ± 4.4	8.5 ± 3.1	0.09

Data are presented as mean ± SD for continuous variables and as number (percentage) for categorical variables. Statistical significance was tested using Student’s *t*-test for continuous variables and Fisher’s exact test for categorical variables. *p* < 0.05 was considered statistically significant.

**Table 2:** Performance comparison of machine learning models.

Model	AUC	ACC	Precision	Recall	F1
Traditional Machine Learning					
Logistic Regression	0.7395 ± 0.1296	0.7110 ± 0.0976	0.5406 ± 0.1056	0.6750 ± 0.2031	0.5897 ± 0.1344
XGBoost	0.6668 ± 0.1061	0.6943 ± 0.0570	0.5107 ± 0.0896	0.4179 ± 0.1559	0.4529 ± 0.1185
Random Forest	0.7084 ± 0.1080	0.7017 ± 0.0401	0.5589 ± 0.1079	0.4107 ± 0.0492	0.4644 ± 0.0228
CatBoost	0.7782 ± 0.0890	0.6860 ± 0.0436	0.5000 ± 0.0777	0.3393 ± 0.1485	0.3881 ± 0.1273
LightGBM	0.6893 ± 0.0892	0.6943 ± 0.0570	0.5107 ± 0.0896	0.4179 ± 0.1559	0.4529 ± 0.1185
KNN	0.7124 ± 0.0569	0.7013 ± 0.1069	0.5466 ± 0.1336	0.6679 ± 0.0956	0.5920 ± 0.1041
Gaussian NB	0.6191 ± 0.1188	0.5393 ± 0.0921	0.3411 ± 0.0512	0.5214 ± 0.3063	0.3752 ± 0.1388
SVM	0.7529 ± 0.1342	0.7417 ± 0.0707	0.6052 ± 0.1279	0.7000 ± 0.2031	0.6239 ± 0.0931
Deep Learning					
MLP	0.7311 ± 0.0635	0.7183 ± 0.0480	0.5750 ± 0.1000	0.4429 ± 0.1571	0.4857 ± 0.1092
ResNet	0.8004 ± 0.0483	0.7823 ± 0.0595	0.6819 ± 0.1009	0.5643 ± 0.1251	0.5643 ± 0.1251
FT-Transformer	0.7987 ± 0.0609	0.7190 ± 0.0930	0.5483 ± 0.1009	0.7000 ± 0.2179	0.6027 ± 0.1351
KAN	0.8250 ± 0.0367	0.7347 ± 0.0576	0.5829 ± 0.0795	0.6179 ± 0.1530	0.5885 ± 0.0959
Advanced Deep Learning Model					
ResKAN-Attention	0.8370 ± 0.0572	0.7503 ± 0.1164	0.6603 ± 0.2122	0.6393 ± 0.0591	0.6333 ± 0.1124

Data are presented as mean ± standard deviation.

## Data Availability

The code is available at https://github.com/LCZ0824/ResKan-Attention (accessed on 4 August 2025) to protect patient privacy, the dataset for this study is not publicly available but can be obtained from the corresponding author upon reasonable request (xy11992@rjh.com.cn).

## Supplementary Materials

The following supporting information can be downloaded at: https://www.mdpi.com/article/10.3390/jcdd13010018/s1, Table S1: Data Preprocessing Parameters; Table S2: Baseline Models Hyperparameter Search Space; Table S3: ResKAN-Attention Training Hyperparameters; Table S4: Knowledge Distillation Parameters; Figure S1: LIME local interpretability analysis; Figure S2: Visualization of learned activation functions within the KAN model; Figure S3: External Validation ROC Curve: ResKAN-Attention Model and Distilled Clinical Formula; TRIPOD Checklist: Prediction Model Development.

## Abbreviations

The following abbreviations are used in this manuscript:

SCAF	Subclinical atrial fibrillation
CIED	cardiac implantable electronic device
KAN	Kolmogorov–Arnold Network
LAD	left atrial diameter
AHRE	atrial high-rate episode
MLP	multilayer perceptrons
ResNet	residual networks
LDH	lactate dehydrogenase
SHAP	Shapley Additive exPlanations
LIME	Local Interpretable Model-Agnostic Explanations
