# Supplementary material for: Subclinical Atrial Fibrillation Prediction in Patients with CIED by a Novel Deep Learning Framework
Source: J Cardiovasc Dev Dis. 2025 Dec 30;13(1):18. doi: 10.3390/jcdd13010018 (PMC12842029; doi:10.3390/jcdd13010018)
Supplement: Supplementary file 1 [file jcdd-13-00018-s001.zip › Figure.pdf]

Supplementary Figure S1. LIME local interpretability analysis. The bar chart in each panel quantifies the contribution of each clinical feature to the final prediction for that specific individual.

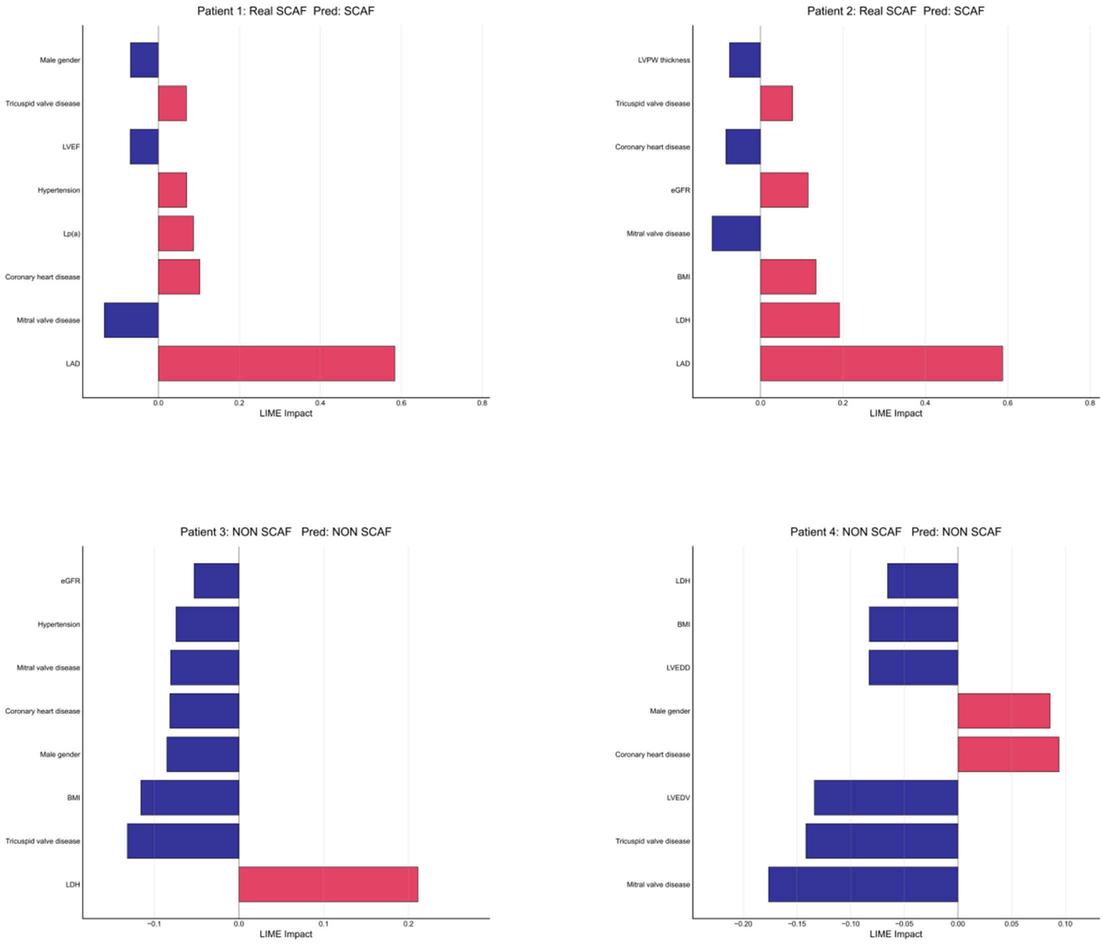

Supplementary Figure S2. Visualization of learned activation functions within the KAN model.

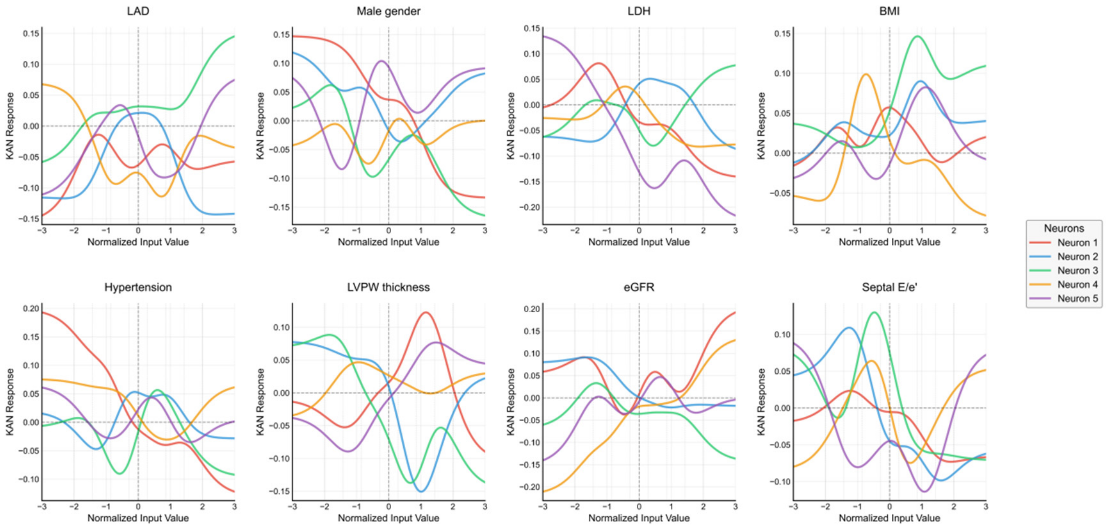

Supplementary Figure S3. External Validation ROC Curve: ResKAN-Attention Model and Distilled Clinical Formula

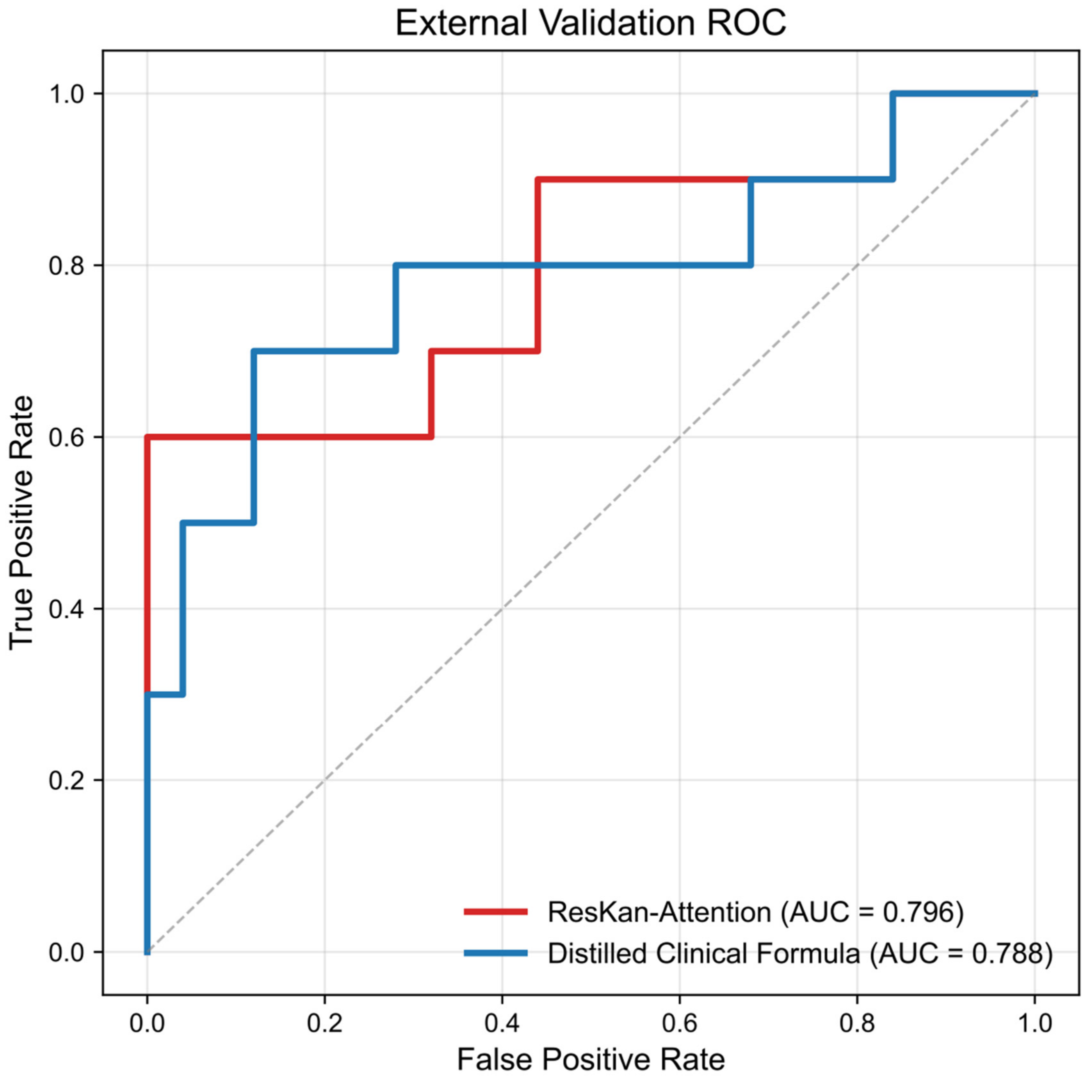

How to use the model

To use the model, clinicians should input 27 routinely available variables, including baseline characteristics, medical history, echocardiographic, and biochemical data. After standard preprocessing, the ResKAN-Attention model outputs the probability of subclinical atrial fibrillation (SCAF) occurrence, ranging from 0 to 1. Higher values indicate higher risk.

For easier clinical application, a simplified formula distilled from ResKAN-Attention,clinicians can directly substitute the corresponding patient values into this formula to calculate the estimated SCAF risk . The model supports early identification and risk stratification of patients prone to SCAF to guide monitoring and preventive management.
