# Supplementary material for: Subclinical Atrial Fibrillation Prediction in Patients with CIED by a Novel Deep Learning Framework
Source: J Cardiovasc Dev Dis. 2025 Dec 30;13(1):18. doi: 10.3390/jcdd13010018 (PMC12842029; doi:10.3390/jcdd13010018)
Supplement: Supplementary file 1 [file jcdd-13-00018-s001.zip › Table.pdf]

Table S1. Data Preprocessing Parameters

Step	Method	Parameter	Value
Missing Value Imputation	IterativeImputer	max_iter	10
		random_state	515
Log Transformation	np.log1p	Applied Features	NT-proBNP, CRP
Standardization	StandardScaler	-	Default
Oversampling	SMOTE	k_neighbors	3
		random_state	515
Cross-Validation	StratifiedKFold	n_splits	5
		shuffle	TRUE
		random_state	515

Table S2. Baseline Models Hyperparameter Search Space

Model	Parameter	Search Space
Logistic Regression	C	[0.1, 1.0, 10.0]
	solver	['liblinear', 'lbfgs']
	penalty	['l1', 'l2']
	class_weight	[None, 'balanced']
	max_iter	2000
SVM	C	[0.1, 1.0, 10.0]
	kernel	['rbf', 'linear', 'poly']
	gamma	['scale', 'auto']
	class_weight	[None, 'balanced']
KNN	n_neighbors	[3, 5, 7, 9]
	weights	['uniform', 'distance']
	metric	['euclidean', 'manhattan']
	p	[1, 2]
Gaussian NB	var_smoothing	[1e-9 to 1e-4]
Random Forest	n_estimators	[50, 100, 200]
	max_depth	[3, 5, None]
	min_samples_split	[2, 5, 10]
	class_weight	[None, 'balanced']
XGBoost	max_depth	[2, 3, 4]
	n_estimators	[30, 50, 70]
	learning_rate	[0.08, 0.1, 0.12]
	reg_alpha	[0, 0.1, 0.3]
LightGBM	max_depth	[2, 3, 4]

	n_estimators	[30, 50, 70]
	learning_rate	[0.08, 0.1, 0.12]
	reg_lambda	[0, 0.1, 0.3]
CatBoost	depth	[2, 3, 4]
	iterations	[30, 50, 70]
	learning_rate	[0.08, 0.1, 0.12]
	l2_leaf_reg	[0, 0.1, 0.3]
MLP	hidden_layers	[[64,32], [64,32,16], [128,64,32]]
	dropout_rate	[0.2, 0.3, 0.4]
	learning_rate	[0.0005, 0.001]
	batch_size	[16, 24]
ResNet	base_dim	[64, 128]
	num_blocks	[2, 3]
	dropout_rate	[0.2, 0.3]
	learning_rate	[0.001]
	batch_size	[16, 24]
FT-Transformer	embed_dim	[32, 64]
	num_heads	[4, 8]
	num_layers	[2, 3, 4]
	dropout_rate	[0.1, 0.2, 0.3]
	learning_rate	[1e-4, 1e-3, 2e-3]
KAN	num_grids	[5, 7, 8]
	dropout_rate	[0.2, 0.3, 0.4]
	learning_rate	[0.0005, 0.0008, 0.001]
	weight_decay	[0.005, 0.01]

Table S3. ResKAN-Attention Training Hyperparameters

Parameter	Value
Optimizer	AdamW
Initial Learning Rate	0.0005
Weight Decay	0.01
LR Scheduler	ReduceLROnPlateau
├─ patience	10
└─ factor	0.7
Loss Function	BCELoss
Batch Size	32
Max Epochs	200

Table S4. Knowledge Distillation Parameters

Parameter	Symbol	Value
Soft Label Weight	α	0.8
Distillation Temperature	T	3
L2 Regularization	λ	0.005
Student Model	-	Logistic Regression
Feature Count	Top-K	10

Loss Function

$$L = \alpha \times KL(T\_soft \,||\, S\_soft) + (1-\alpha) \times BCE(y\_true, y\_student) + \lambda||\theta||^2$$
